# Recent and Projected Changes in Global Climate May Increase Nicotine Absorption and the Risk of Green Tobacco Sickness

**DOI:** 10.21203/rs.3.rs-3830008/v1

**Published:** 2024-02-02

**Authors:** Lewis Ziska, Robbie Parks

**Affiliations:** Columbia University; Columbia University Mailman School of Public Health

**Keywords:** Climate Change, Green Tobacco Sickness, Nicotine Absorption, Tobacco

## Abstract

**Background.:**

Dermal transfer of nicotine during tobacco harvest can increase green tobacco sickness (GTS), characterized by nausea, vomiting, headache and dizziness. Rainfall and temperature are established etiological factors known to increase prevalence of GTS. We aimed to analyze recent and projected trends in these factors for major tobacco growing regions to assess potential exacerbation in GTS occurrence.

**Methods.:**

We analyzed climate parameters, including trends in temperature and precipitation metrics during the tobacco harvest period for Southern Brazil; Yunnan Province, China; Andhra State, India; and North Carolina, USA (~50-year period). We applied Shared Socio-economic Pathways (SSPs) based scenarios for CMIP6, (SSPs of 1–2.6, 3–7.0 and 5–8.5 from 2020 to 2100). Established protocol for nicotine dermal patches and temperature was used as a proxy to estimate potential nicotine absorption with rising temperature.

**Results.:**

For three locations, cumulative maximum temperatures during harvest season and temperature extremes increased significantly since the 1970s. For all locations, cumulative rainfall during the harvest season also rose. Projected maximum temperatures for the harvest season increased at SSP 3–7.0 and 5–8.5 projections through 2100 for all locations. Estimates of nicotine skin absorption with rising temperature indicate significant increases for both recent changes (since the 1970s) in three of the four locations, and for all locations for the SSP projections of 3–7.0 and 5–8.5 from 2020 to 2100.

**Conclusions.:**

This study across multiple continents, highlights a potential link between recent and projected anthropogenic change and potential increases in GTS risk. Under SSP 5–8.5, nicotine absorption could increase by ~50% by the end of the century, which may have widespread impacts on the incidence of GTS, especially among younger tobacco workers.

## Introduction

Documenting and addressing the interactions between anthropogenic climate change and human health remains a vital challenge for the remainder of the 21st century^1^. Changes in the increase and variability of temperature, humidity and precipitation, both recent and projected, are likely abiotic forcers with respect to human health, from heat-related illness to air quality^2^. Agricultural workers, characterized by long hours of high labor exertion in outdoor environments, may be especially vulnerable to climate related hazards^2–4^. Such vulnerability, in turn, may also have adverse effects relative to agricultural production^5^.

Field grown tobacco prior to harvest is known as “green tobacco”. During tobacco harvest, workers who remove and collect leaves may suffer from an occupational illness known as “green tobacco sickness” (GTS)^6^. GTS results from dermal absorption of nicotine from the leaves through handling, leading to nicotine poisoning; symptoms include chills, diarrhea, weakness, salivation and dizziness. The onset of GTS varies from 3–17 hours following exposure and can last from 1 –3 days. Initial responses include cessation of work, change of clothing, showering, fluid intake and rest; extreme cases can result in intravenous rehydration, anti-emetics and dimenhydrinate^7^. Prevalence can vary^8^, with an estimate of approximately 24.2 percent among Latino farmworkers^9^. Current global estimates for children (12–18 y.o.) working in tobacco would indicate that over 300,000 children suffer from GTS in a given season^10–12^

Two abiotic metrics are widely recognized as determining the extent of nicotine absorption and GTS. First, absorption is water dependent and increases in conjunction with rain occurrences, dew and sweat; second, the combination of high ambient temperatures and physical labor results in greater perspiration, surface blood flow and increasing dermal absorption^13–15^. McKnight and Spiller^16^ estimate that moisture on tobacco leaves may contain as much as 9 mg of dissolved nicotine per 100 mL of dew, roughly equivalent to the nicotine content of six average cigarettes^13^. On a humid day, especially after a recent rain, the average field worker may be exposed to as much as 600 mL of dew^13^, the equivalent of smoking 36 cigarettes.

Although the physical environment (temperature and rainfall) is well documented in conjunction with the etiology of nicotine absorption and GTS occurrence, the role of anthropogenic climate change relative to these metrics has not, to our knowledge, been evaluated. Here, we provide the first such assessment regarding recent climate trends (~ 50 years), and future climate projections for these variables specific to the tobacco harvest season for diverse tobacco growing regions. It is important to confirm for these locations the degree and or extent of shifts in maximum temperatures and precipitation patterns associated with anthropogenic climate change; and to begin an initial assessment regarding any potential exacerbation of nicotine absorption and GTS.

## Methods

### Locale.

As of 2021, the top five tobacco producing countries globally are China, India, Brazil, Indonesia and the United States. As long-term meteorological data for Indonesia was not available, we examined production statistics within the remaining countries https://www.fao.org/land-water/databases-and- software/crop information/tobacco/en/; focusing on regions within Southern Brazil (States of Parana, Santa Catarina and Rio Grande do Sol); China (province of Yunnan), India (state of Andhra Pradesh) and the U.S. (eastern North Carolina) where tobacco has been widely grown. Harvest, or “priming”, the gathering of leaves for curing, the time of greatest nicotine exposure, varied by location, estimated days of year (DOY) were 1–66; 213–250; 32–90 and 196–220 for Brazil, China, India and North Carolina, respectively. Leaf collections were not observed at any given location.

### Meteorological Data.

For each harvest season period, we obtained daily temperature and rainfall data from at least 4 weather stations located within each tobacco growing region. These data were obtained using a software program developed by Texas A&M University (College Station, TX, USA) which collates all online weather station data at a global level^17^. For harvest season data metrics were applied that would be pertinent to nicotine transfer (1972–2022); i.e, cumulative maximum daily temperatures; extreme temperature occurrence (site specific); cumulative rainfall and number of rainy days.

For projected climatic changes, the Common Management Information Protocol, IPCC sixth assessment report, (CMIP6) was used for scenario runs through the Royal Netherlands Meteorological Institute (KNMI) Climate Explorer (https://climexp.knmi.nl/), a web-based tool to investigate climate data and generate statistics (currently hosted by the World Meteorological Organization (WMO) Regional Climate Centre).We downloaded the CMIP6 mean values for temperature and precipitation for each of three climate change projection scenarios (ssp126, ssp370, ssp585) that correspond respectively to three scenarios: a gradual but consistent shift toward sustainability and greenhouse gas (GHG) reduction; more nationalistic, with low international priority for addressing environmental, including GHG, limits; limited GHG regulation, and lack of participatory activities, continuation of energy intensive lifestyles, inclusion of geo-engineering if necessary^18^. We then summarized for each tobacco growing location and harvest dates, the maximum temperature and precipitation projections through 2100. Note that while trends produced by CMIP6 are acceptable to analyze, the absolute values by themselves are estimates^19^.

### Estimates of Nicotine Absorption.

Numerous studies and analyses have shown that absorption of compounds placed on the skin is accelerated with elevated temperatures^20–21^. Heat accelerates skin blood perfusion, which can accelerate chemical transfer through the skin. At present, there exist no data specific to nicotine absorption by temperature from tobacco leaves. However, there are data regarding the role of temperature and modeled estimates of nicotine absorption in therapeutic dermal patches^22–24^. It can be argued that such a temperature response is dependent on continuous dermal contact with the patch over^8–10^ hours. Yet, during harvest, tobacco leaves as they mature are picked by hand, usually placed under the arm, and can be in direct contact with skin, (or with an article of clothing that is saturated with sweat, nicotine is water soluble), for 10–12 hours. A dermal nicotine patch is 22.5cm^2^, and can contain from 7–21 mg of nicotine; a harvested tobacco leaf at 4% nicotine (tobacco leaves at harvest vary from 3–5%) would contain ca 5.7 mg for the same surface area^25^. We used the relationship between dermal nicotine patches and temperature as a proxy to estimate relative changes in tobacco nicotine absorption with temperature^24^.

### Statistical Analysis.

We aimed to assess long-term (~ 50 year, ~ 1975–2022) trends for cumulative maximum daily temperatures and cumulative rainfall over the harvest season, changes in the number of high temperatures above a threshold and the number of days of rain. To assess potential temporal shifts, seasonal regressions (slopes) of variation were determined for each metric over time for all 4 locations at the p < 0 05 and p < 001 levels. To aid the reproducibility of our study, the meteorological data from all weather stations at a given location will be available on GitHub. Similarly for future projections regarding maximum daily temperature and average daily rainfall for each location (2020–2100) using the SSP projections from CMIP6, linear regression over time was used as a statistical variable.

## Results

**Figure 1:**
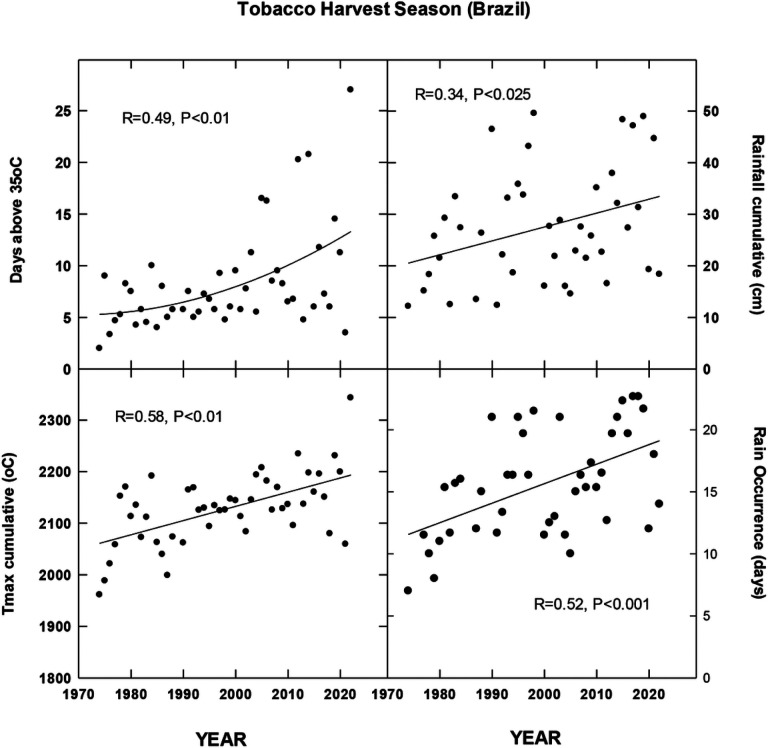
Temporal temperature and rainfall changes in southern Brazil, ~past 50 years. See Methods for additional details.

**Figure 2:**
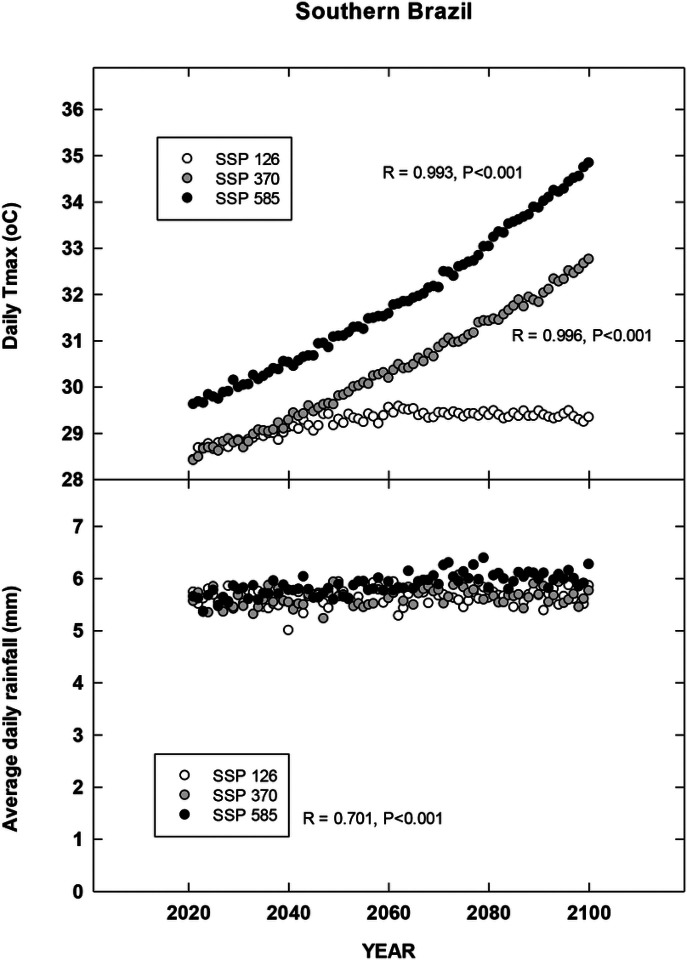
Projected Changes in maximum temperature and average rainfall for southern Brazil. See Methods for additional details.

**Figure 3:**
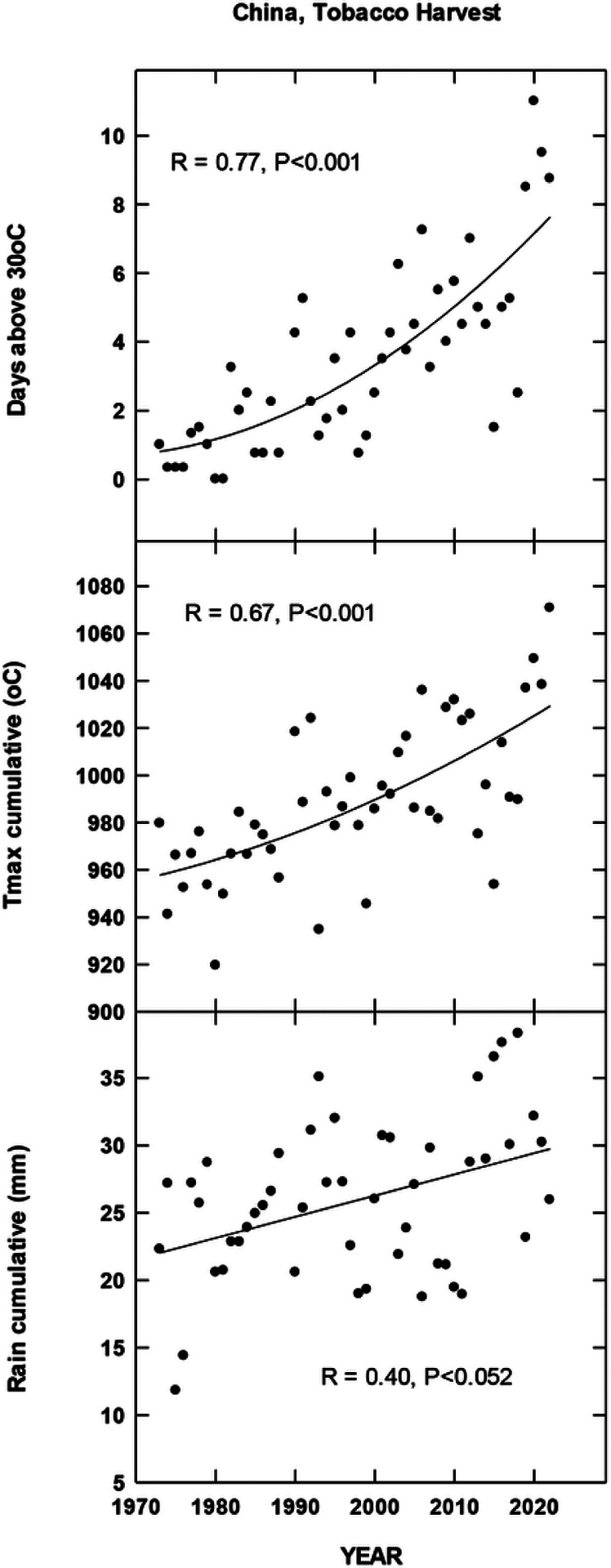
Temporal temperature and rainfall changes in Yunnan Province, China, ~past 50 years. See Methods for additional details.

**Figure 4:**
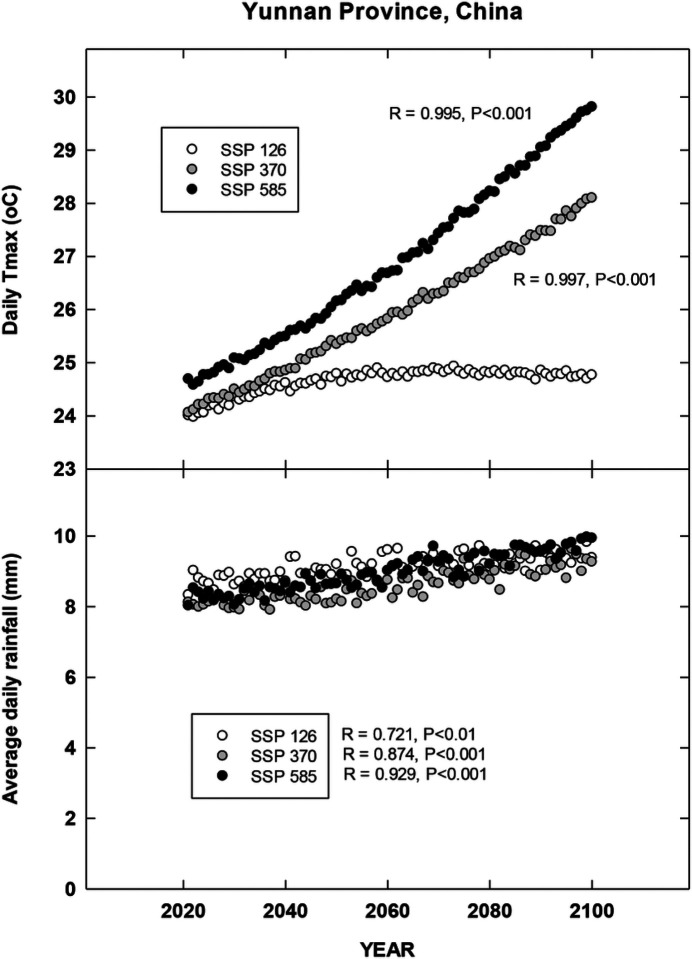
Projected Changes in maximum temperature and average rainfall for Yunnan Province, China. See Methods for additional details.

**Figure 5:**
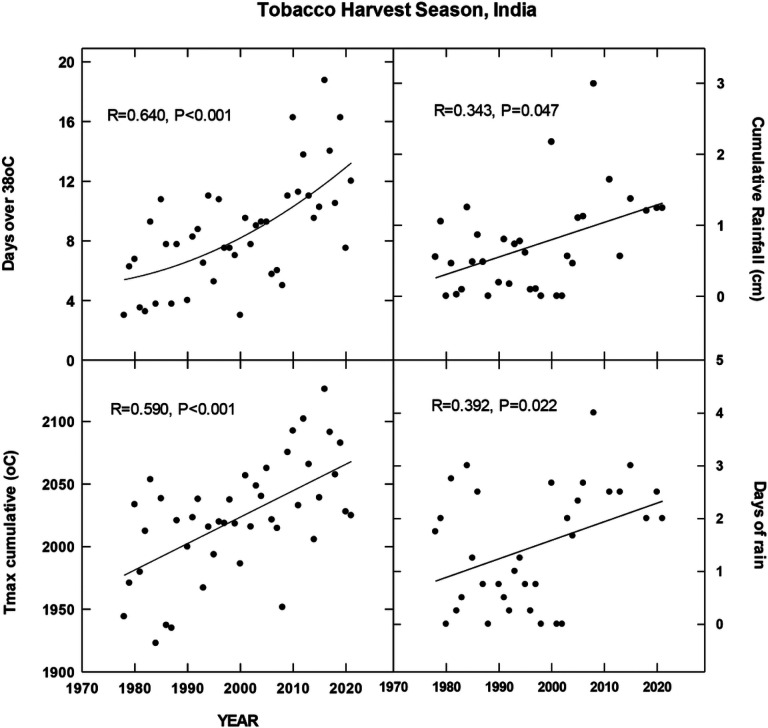
Temporal temperature and rainfall changes in Andhra Pradesh, India, ~past 50 years. See Methods for additional details.

**Figure 6:**
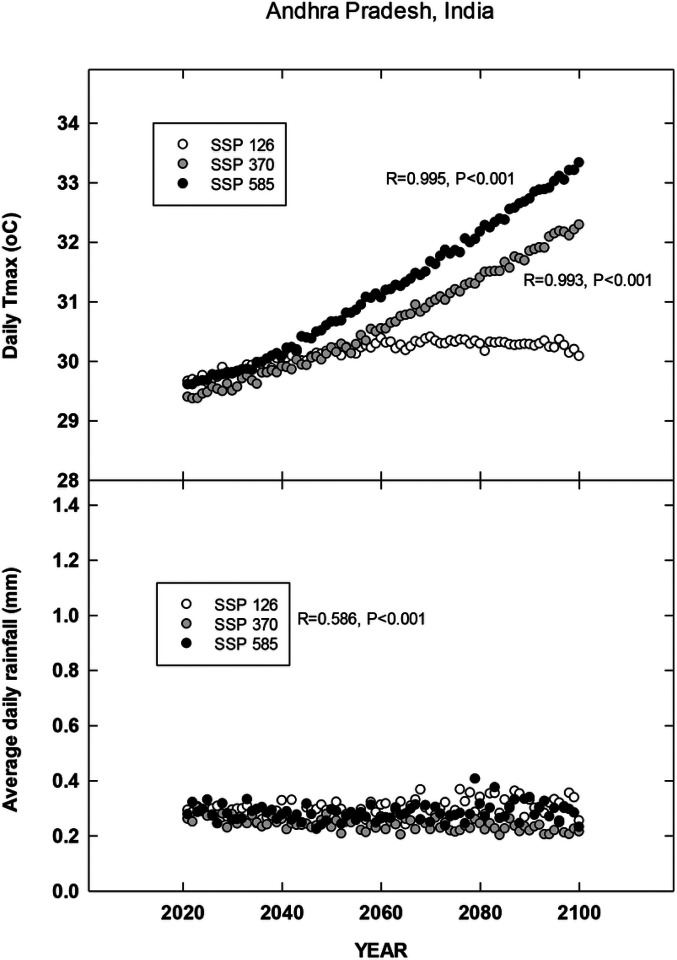
Projected changes in maximum temperature and average rainfall for Andhra Pradesh, India. See Methods for additional details.

**Figure 7:**
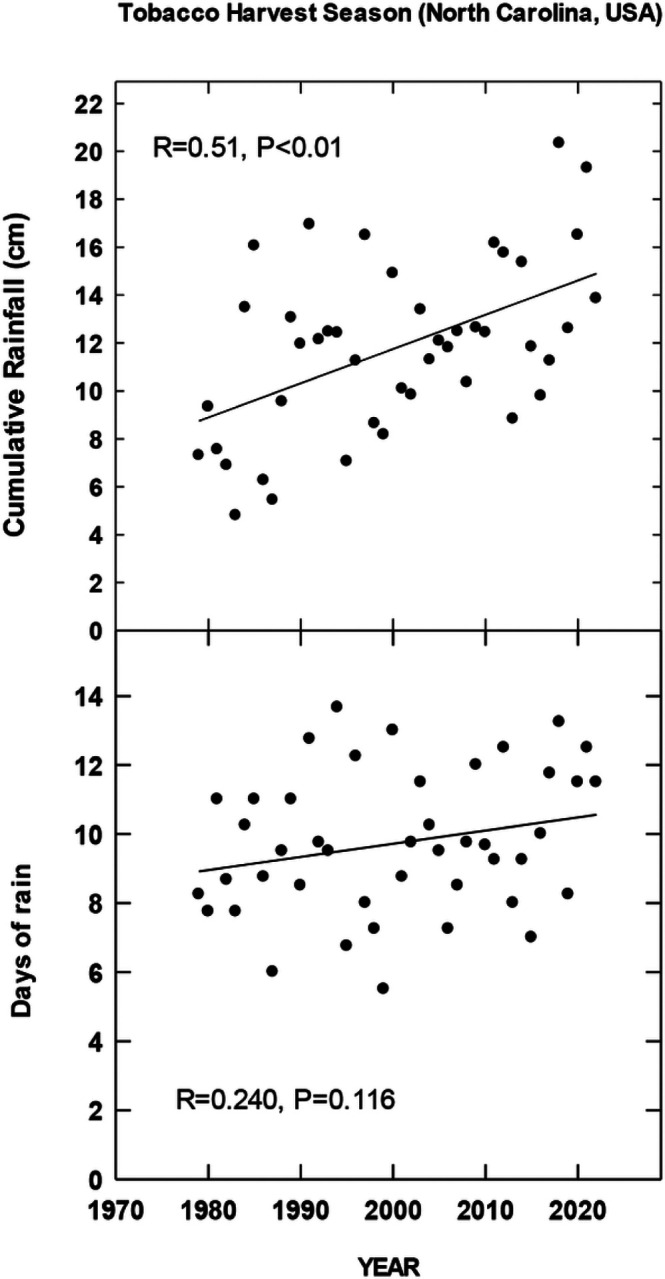
Temporal temperature and rainfall changes in North Carolina, USA, ~past 50 years. See Methods for additional details.

**Figure 8:**
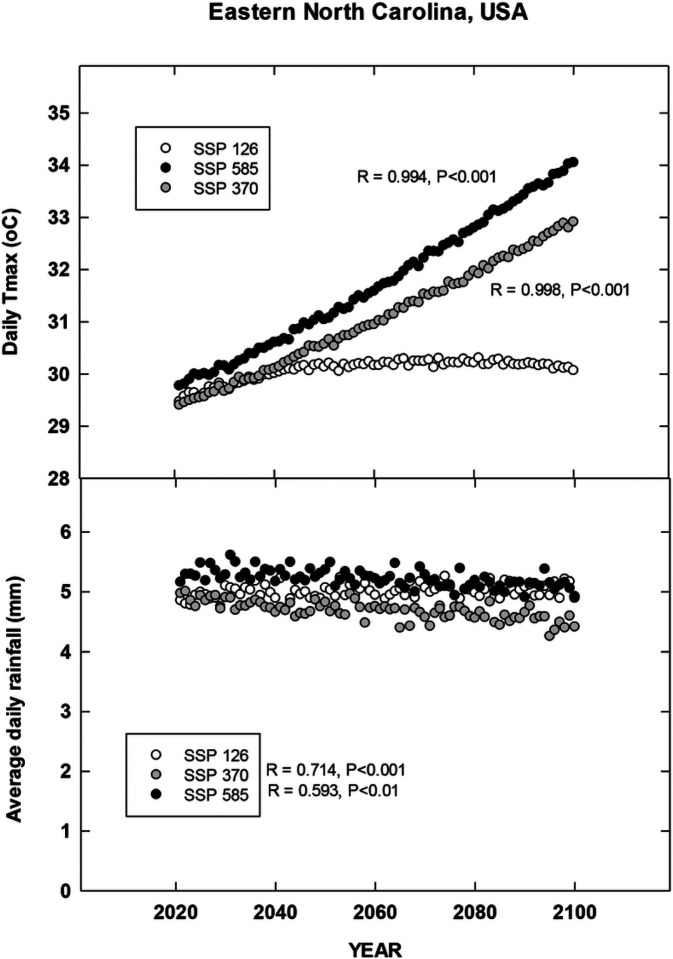
Projected changes in maximum temperature and average rainfall for Eastern North Carolina, USA. See Methods for additional details.

For each country location, first order regressions were done to ascertain any recent temporal shifts for cumulative maximum daily temperatures, occurrence of extreme temperatures (days at 35, 30, 38 and 35°C for Brazil, China, India and North Carolina, respectively), cumulative rainfall (cm) and occurrence of rain (days). Overall, we found significant increases in all four metrics for Brazil for the period 1972–2022 during the harvest season (Fig. 1). CMIP6 projections for Brazil indicate significant increases in daily maximum temperatures for the SSP 370 and 585 scenarios. There is a trend for increasing average daily rainfall for all CMIP projections but only significant for 585 SSP (Fig. 2). For the Yunnan province in China, increases in days above 30°C, cumulative increases in maximum temperature during the tobacco harvest season and cumulative rainfall were all observed from 1972 through 2022 (Fig. 3). Future projections for this region indicated significant increases in average daily maximum temperatures through 2100 for SSP 370 and SSP 585; in addition, average daily rainfall was significantly increased for all SSP scenarios (Fig. 4). For the Andhra Pradesh state in the southern coastal region of India, significant increases (1978–2021) were observed in the number of days over 38°C, increases in the cumulative maximum temperatures and rainfall, as well as increases in the number of days of rain (Fig. 5). Modelled increases for this region indicate significant shifts in daily maximum temperatures (SSP 370 and SSP 585); forecasts for daily rainfall were equivocal; however, a significant increase was observed for SSP 370 (Fig. 6). In contrast to other locations, eastern North Carolina, showed no significant recent changes in temperature metrics (1977–2022); however significant increases in cumulative rainfall were noted (Fig. 7). CMIP6 projections for this location indicated significant increases in maximum temperatures for SSP 370 and 585. A downward trend (significant for SSP 370 and SSP 585) was observed for average daily rainfall through to 2100 (Fig. 8).

Overall, for three of the four locations, there is evidence of recent climatic trends associated with temperature and rainfall metrics and one location, North Carolina, where only cumulative rainfall during the harvest season was noted. For modelled projections however, significant increases in maximum temperature were observed for moderate and high-end scenarios (SSP 370 and SSP 585, respectively) for all locations; increases in average daily rainfall were estimated to increase for Brazil and China, trends for India and North Carolina were uncertain.

Although these conditions *in toto* are likely to exacerbate GTS, one metric, increases in maximum air temperature (T_max_), can be related by proxy to increases in nicotine absorption. Recent temperature increases (1975–2022) show a slight, but significant, potential increase in absorption for 3 of the 4 locations (**Table 1**); similarly larger temperature increases associated with SSP 370 and 585 for century’s end show larger potential for greater nicotine absorption (**Table 1**), and increased GTS, but for all locations.

## Discussion

A rapidly warming climate poses a unique and growing threat to public health. The health of outdoor agricultural workers is considered among the most vulnerable^26^.

Tobacco production risks include a number of occupational hazards, most notably nicotine poisoning, the dermal absorption (“dose”) of nicotine from the tobacco leaves, is acknowledged as the cause of GTS^8, 13–15^. The extent of this illness among tobacco workers varies considerably^13–15^; however, it is important to emphasize that GTS is not well documented and often underreported because those who experience GTS do not always recognize the basis for their sickness^6,7^. As such they are often left voiceless, underrepresented in public health assessments.

In this study, the temporal climate data for recent (since the 1970s) changes in temperature and rainfall specified significant increases for three of the four locations studied. Projected changes in the SSPs that reflect moderate and high mitigation and adaptation challenges to anthropogenic climate change (SSP 370 and 585) indicated significant increases in maximum temperature for all locations studied for the remainder of the century. Note that we focused on harvest periods for each location. This is a time of maximum dermal absorption, as harvesting is done manually workers will hold cut leaves close to their body, where nicotine rich leaf exudate or leaf moisture can soak clothing, increasing dermal absorption. It has been estimated that harvesting involves the greatest risk of GTS occurring followed by barning (packing it in storage in a barn)^8^. Also note that the locations chosen for this study are representative of major tobacco growing regions globally. Any increase in rainfall, or temperature as shown here, will increase nicotine exposure, and the risk of GTS. Such risks would be exemplified for example, by increases in temperature induced dermal absorption of nicotine (e.g., **Table 1**). As tobacco harvest occurs during late summer, heat will increase skin permeability, body fluid circulation, blood vessel wall permeability and chemical absorption^27^ It has been shown that skin blood flow is maximal when skin surface temperature nears 42°C as heat is being shed dermally^24^.

However, there are a number of caveats. The etiology of GTS is complex. If dermal transfer (“dose”) increases, there are a number of factors that influence health outcomes (“response”). For example, experience, age, degree of skin exposure can affect percutaneous dermal absorption and the occurrence of GTS^15–16^. The Occupational Health and Safety Office (OSHA, (https://www.osha.gov/green-tobacco- sickness) has stressed that new workers may have a lower nicotine tolerance relative to previous workers, they may also be less aware of GTS and personal protection equipment (PPE). Children and adolescents with younger skin may be more sensitive to chemical exposures, more likely to suffer if GTS occurs, and may, in turn, experience more serious health effects than adults^28–29^. Tobacco farming is well recognized globally as a source of employment for children. For example, while the risks of tobacco farming are acknowledged by the U.S. government, it is still legal for kids, beginning at age 12, to work on a tobacco farm (of any size) with parental permission. For southern Brazil, in a cross-sectional demographic study, 99 young people were interviewed at 79 family farms. For those interviewed, ~ 60% were 16–17 years old, and 51.5% were male. During their lifetime, ~ 25% reported GTS, and 3% pesticide poisoning^30^.

The participation of children adds additional health concerns in the context of climate change beyond GTS risks. Child workers may be especially liable to climate change influences especially extreme heat^31–32^. They are less capable of temperature regulation, especially during high ambient temperatures, and dependent on adults for environmental protection^8^. Vomiting, a common GTS response, can also exacerbate water loss and contribute to the risk of heat exposure for child workers. Overall, the projected changes in maximum temperature shown here are likely to intensify the effect of nicotine absorption and GTS on child tobacco workers.

Much remains to be learned regarding the epidemiology of GTS. At present the incidence, extent and severity of GTS has not been well characterized. Often the only data for GTS is location specific and primarily descriptive^6^. Yet, as the tobacco industry expands production to the developing world, exposure to GTS will increase, and the need to understand the health consequences and resulting treatments, through a climate change lens, imperative.

However, the data presented, both with respect to quantification of recent environmental changes and modelling of future temperature and rainfall metrics, strongly suggest an increase in the dermal transfer of nicotine. The proxy metric of nicotine absorption used here can help to provide a specific quantitative risk of GTS relative to climate change, but additional details related to age, smoking history, etc. are needed. Such an effort to characterize absorption will also need to consider integration of all climate parameters as well as other variables such as location, demographics, availability of PPE, and other factors. These recent and projected climate data may prove useful for developing nicotine absorption and future GTS exposure estimates that may be especially consequential in the context of children’s health. Such information is essential to improve our overall understanding of the prevalence, intensity and distribution of global GTS occurrence.
